# Rheostats, toggles, and neutrals, Oh my! A new framework for understanding how amino acid changes modulate protein function

**DOI:** 10.1016/j.jbc.2024.105736

**Published:** 2024-02-08

**Authors:** Liskin Swint-Kruse, Aron W. Fenton

**Affiliations:** Department of Biochemistry and Molecular Biology, The University of Kansas Medical Center, Kansas City, Kansas, USA

**Keywords:** protein evolution, protein engineering, protein function, protein stability, personalized medicine

## Abstract

Advances in personalized medicine and protein engineering require accurately predicting outcomes of amino acid substitutions. Many algorithms correctly predict that evolutionarily-conserved positions show “toggle” substitution phenotypes, which is defined when a few substitutions at that position retain function. In contrast, predictions often fail for substitutions at the less-studied “rheostat” positions, which are defined when different amino acid substitutions at a position sample at least half of the possible functional range. This review describes efforts to understand the impact and significance of rheostat positions: (1) They have been observed in globular soluble, integral membrane, and intrinsically disordered proteins; within single proteins, their prevalence can be up to 40%. (2) Substitutions at rheostat positions can have biological consequences and ∼10% of substitutions gain function. (3) Although both rheostat and “neutral” (defined when all substitutions exhibit wild-type function) positions are nonconserved, the two classes have different evolutionary signatures. (4) Some rheostat positions have pleiotropic effects on function, simultaneously modulating multiple parameters (*e.g.*, altering both affinity and allosteric coupling). (5) In structural studies, substitutions at rheostat positions appear to cause only local perturbations; the overall conformations appear unchanged. (6) Measured functional changes show promising correlations with predicted changes in protein dynamics; the emergent properties of predicted, dynamically coupled amino acid networks might explain some of the complex functional outcomes observed when substituting rheostat positions. Overall, rheostat positions provide unique opportunities for using single substitutions to tune protein function. Future studies of these positions will yield important insights into the protein sequence/function relationship.

Many applications, from protein engineering to interpreting differences in genome sequences, require accurately predicting the effects of amino acid substitutions. Unfortunately, most prediction algorithms have low success rates, indicating that some fundamental feature(s) of the sequence/function relationship have not yet been accounted for (1). The purpose of the current review is to briefly describe the limitations and biases that have historically affected prediction algorithms and then to review the past decade’s experimental studies that aimed to rectify deficiencies in the data used to build prediction algorithms. These studies have revealed several surprising features of an important class of “rheostat” protein positions, for which substitutions modulate protein function. In the future, it will be critical to understand these features at the biophysical level to improve predictions about functional outcomes.

As a prelude to these topics, it is useful to first define “protein function”. In this article, “function” refers broadly to any molecular event performed by a folded protein (with the exception of intrinsically disordered proteins, discussed below) and *not* including changes in protein stability. When particular examples are reviewed, biochemical descriptors are provided (*e.g.*, binding affinity, K_d_). We further note that a complete description of “protein function” usually requires multiple biochemical parameters. For example, full characterization of an enzyme includes binding affinities for all substrates and turnover numbers for forward and reverse reactions. Ideally, one would monitor all parameters associated with all of a protein’s functions, but this is often impractical. To circumvent this limitation, some studies monitor substitution outcomes using biological assays that are sensitive to changes in multiple functional parameters; these assays are often also sensitive to changes in protein concentration, folding, or stability. The tradeoff is that biological readouts are less informative for learning how proteins actually work; biochemical and biophysical assays that quantify discrete functional aspects (or stability) provide higher-resolution insight. Fortunately, even with their respective limitations, both biochemical and biological studies have been useful for elucidating the characteristics of rheostat positions.

### Historical limitations of prediction algorithms and the existence of rheostat positions were identified by a “semiSM” experimental design

Nearly all prediction algorithms incorporate the same input and three underlying assumptions about protein substitutions. Their common input is the use of a multiple sequence alignment (MSA) for each protein of interest. Within each MSA, sequence variation is treated as a “natural mutation experiment”: If a particular amino acid is present in a particular MSA column (amino acid position) that amino acid is presumed to allow protein function to occur. A corollary is that the absence of a particular amino acid from a position’s column is often inferred to indicate that the substitution is detrimental.

The three additional assumptions that underlie prediction algorithms are the culmination of decades of substitution experiments and analyses of protein families. The first two “rules” are even taught to undergraduates: (i) If a particular position in a protein is “important”, most amino acid substitutions are expected to be catastrophic. (ii) A few side chains might be tolerated (allow function) if they are chemically similar to the wild-type amino acid; these alternative amino acids are often observed in an MSA column. For example, if a side chain hydroxyl is required in an active site, all homologs contain either a serine or a threonine at that position. The third (albeit often implicit) assumption arises because an MSA is used as input: (iii) Information about an amino acid change at one position in one protein can be extrapolated to the analogous position in all other homologs.

Unfortunately, these assumptions about substitution outcomes have been influenced by an under-appreciated bias: Before ∼2012, most substitution studies were performed at evolutionarily conserved positions (2). At these positions, many changes are indeed catastrophic and “toggle off” function, consistent with the canonical rules. (Indeed, historic studies of evolutionarily-conserved positions provided the observations used to generate the canonical rules.) In contrast, changes at nonconserved positions were largely overlooked, despite their key roles in paralog evolution. Thus, a decades-long study of LacI and other members of the LacI/GalR family was carried out to test whether the canonical rules described above are appropriate for understanding amino acid substitutions at nonconserved positions (3, 4, 5, 6, 7, 8, 9, 10, 11, 12). A key design feature of these (and subsequent) studies was to substitute each position with ∼10 to 14 different, randomly chosen amino acids—a semi-saturating mutagenesis approach (semiSM). Protein variants were then assessed with functional assays.

This semiSM approach revealed a novel class of “rheostat” positions (*e.g.*, Fig. 1; (11)) that would not have been readily apparent from other strategies used to identify functionally relevant positions, such as alanine scans, directed evolution, and ancestral reconstruction. Furthermore, substitutions at the rheostat positions did not follow *any* of the canonical substitution rules listed above: First, the protein variants derived from semiSM of each position exhibited a wide range of functional outcomes. For example, ten substitutions at one LacI position exhibited DNA binding affinities that sampled nearly three orders of magnitude (4); follow-up studies with other positions in LacI and its homologs identified many positions with this behavior (5, 6, 7, 8, 10, 11). Second, neither side-chain chemical similarities nor evolutionary frequency explained the rank order of substitution outcomes observed for individual positions (11); these findings were not unique to the LacI/GalR homologs – other studies also noted such puzzling results (13, 14, 15). Third, a specific substitution in one homolog did *not* cause the same functional change in other homologs (5, 6, 7, 8, 10, 11, 12). Fourth, ∼10% of substitutions at rheostat positions showed a gain-of-function (11); a similar percentage of gain-of-function variants was identified in a recent search of the variants in the Human Gene Mutation Database (16). Finally, separately predicting the outcomes of amino acid changes for both rheostat and toggle positions, using 16 mathematically distinct computer algorithms, was successful for toggle positions but *not* rheostat positions (1).

**Figure 1:**
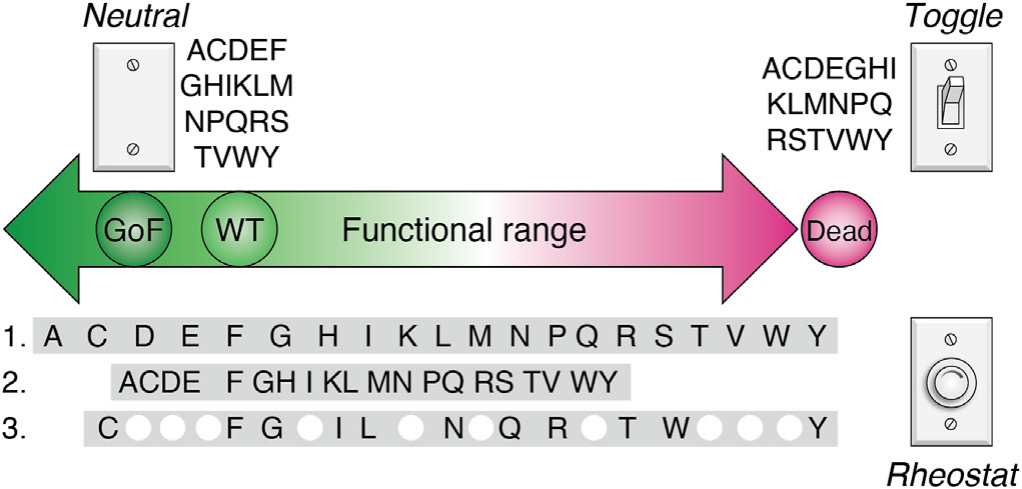
**Examples of two conventional and three possible rheostat substitution outcomes.** The overall substitution behaviors of neutral (*top left*), toggle (*top right*), and rheostat (*bottom*) positions can be revealed by a semiSM approach. The *double arrow* in the *middle* of this figure represents the total range of possible functional outcomes that can be measured for a hypothetical functional parameter; *circles* are used to indicate the relative locations of wild-type (WT) and “dead” variants; the *arrow* extends to the *left* of WT to indicate gain-of-function variants (“GoF”). Five possible outcomes are shown: (*upper left*) neutral position, all substituted variants cluster with the wild-type phenylalanine; (*upper right*) toggle position, all substitutions are catastrophic; (below the *arrow*) three versions of a rheostat position, variants exhibit a range of functional outcomes. The three rheostat examples shown here exhibit (i) an “ideal” rheostat behavior, for which the 20 possible substitutions evenly sample the full functional range; (ii) a “partial range” behavior, for which 20 substitutions sample more than half but less than the full functional range; and (iii) a “sampled range” behavior, for which fewer than 20 substitutions are sufficient to sample more than half of the full functional range (white circles represent untested amino acids). These five examples are also used in Figure 2 to exemplify the modified histogram analysis for quantitative neutral, toggle, and rheostat assignments.

Clearly, a better understanding of this under-appreciated class of rheostat positions provides one avenue for improving predictions of functional change. In the years since the LacI/GalR studies, semiSM studies of multiple model proteins have been performed to (i) assess which structural classes of proteins have the potential to contain rheostat positions; (ii) assess the prevalence of rheostat positions within individual proteins; (iii) assess whether rheostat positions are associated with a particular type of “nonconservation”; (iv) identify nonconserved positions that are *not* important to function (*i.e.*, neutral positions, which accept most substitutions without consequence) and are thus distinct from rheostat positions; (v) explore the range of functional parameters that can be affected by substitutions at rheostat positions; and (vi) glean information about potential conformational and dynamic changes that give rise to the non-canonical substitution outcomes.

### Rheostat, toggle, and neutral positions can be objectively assigned

In addition to revealing to the novel class of rheostat positions, a comprehensive study of LacI/GalR homologs (11) identified positions with the two conventional substitution outcomes illustrated in Figure 1: First, as mentioned above, the canonical rules predict that substitutions at important amino acid positions exhibit a “toggle” outcome; that is, the function would either be “on” or “off”, depending on which side chain is present. Second (and at the other extreme), nonconserved positions are often expected to be “neutral”; that is, substitutions at these positions have no functional effect (with the possible exceptions of proline and glycine, which alter the properties of the peptide backbone).

Assigning rheostat, toggle, and neutral outcomes to each position in the LacI/GalR study was readily accomplished by visual inspection of the data, which spanned five orders of magnitude. In subsequent studies, the need for quantitative assignments became clear and a modified histogram analysis (“RheoScale”) was developed (Fig. 2; (17)). Using this scoring system, a quantitative definition of rheostat positions was based on their sets of substitutions sampling at least half of the possible functional range. This scoring system is further described in the legend to Figure 2 and was used to mathematically demonstrate that a semiSM approach can obviate the expense of site-saturating mutagenesis of an individual amino acid position (SSM; all 20 amino acids assessed at each position) (17).

**Figure 2:**
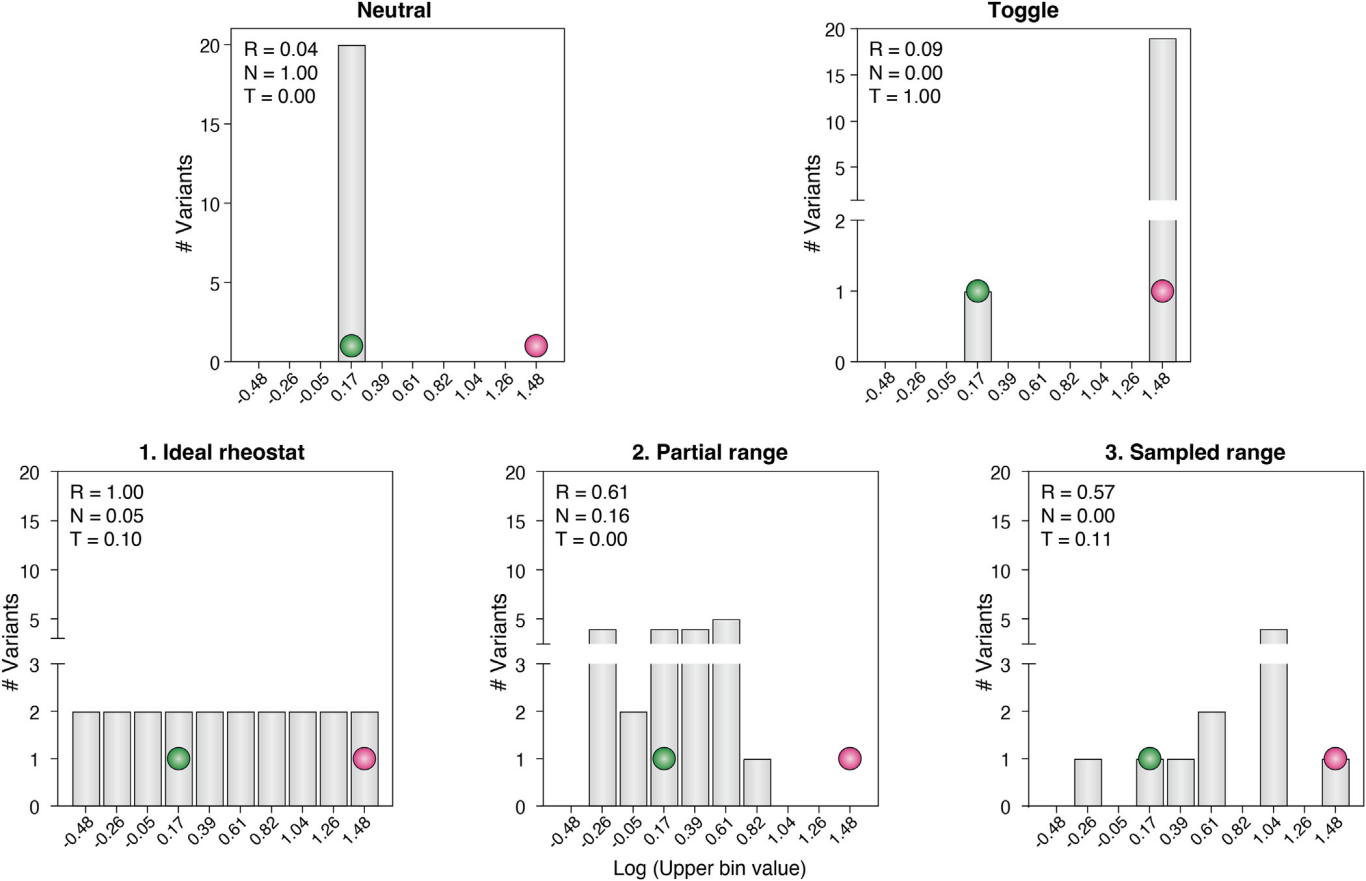
**Example histogram analyses used to derive rheostat (R), toggle (T), and neutral (N) scores for the examples in**Figure 1**.** When functional outcomes are known for a set of substitutions at a single position (either semiSM or SSM), modified histogram analysis data can be used to classify each amino acid position as a neutral, rheostat, or toggle position. This analysis can be used for any quantitatively assessed functional parameter (biochemical or biological), as well as measures of protein stability. Two features are important for this analysis: the overall range and the number of bins. The histogram range is derived from the entire substitution set, which preferably includes data for multiple positions. As shown by the *arrow* in Figure 1, one end of the range is defined by a “dead” variant that lacks detectable activity (*magenta dot*). The other end is defined by the “best” observed function; this could be the wild-type variant (*green dot*), although many studies reported to date have identified variants with function better than wild-type. Considerations for choosing the bin number are described in (17). Empirically, ten bins work well for most datasets; sparse datasets can be analyzed with as few as four bins (*e.g.*, LacI in (33)). Analyses score bin occupancy as either 0 (unoccupied) or 1 (occupied by any number of substitutions); to account for experimental error, intermediate bins can be weighted more heavily than those adjacent to the WT; the occupancy of all bins is used to calculate rheostat, toggle, and neutral scores, each of which ranges from 0 to 1. Here, the examples in Figure 1 were simulated with a WT functional value of 1, a “best” value of 0.2, and a “dead” value of 30. Histogram bins are labeled with the log of the value of their upper limit. Four examples were simulated with n = 20 variants; the third rheostat example was simulated with ten variants. The R, N, and T scores associated with each example are shown within the plots. The neutral score reflects the percent of non-WT substitutions that exhibit WT-like function; factors relevant to a significance threshold of 0.7 are in (31); a truly neutral position must be neutral for all parameters that can be measured (31). The toggle score reflects the fraction of substitutions that result in “dead” protein; factors relevant to a significance threshold of 0.64 are in (1). The rheostat score reflects how well a set of substitutions samples the observed range; the significance threshold of 0.5 can be simplistically interpreted as “the set of substitutions samples at least half of the functional range” (17). The sampled range can either be continuous (*e.g.*, “ideal rheostat” and “partial range”) or discontinuous (*e.g.*, “sampled range”).

Among its advantages, RheoScale analyses avoid the false dichotomy of classifying each position as either “WT-like” or “deleterious”; when dichotomous categories are used to classify either position phenotypes or substitution outcomes, results with intermediate values are often ignored (*e.g.*, (18, 19)). Furthermore, Rheostat scores (i) provide a convenient summary of the SSM datasets that are usually shown as large heatmaps (*e.g.*, (20, 21, 22, 23, 24, 25)) but are difficult to digest or compare to structures, yet (ii) retain the position-specific information that is obscured in histograms that aggregate all substitution data for all positions (*e.g.*, (23, 25, 26, 27)). A “weighted score” was recently used to quantify inactive variants (27) and is similar to the “toggle score” of RheoScale, but such a score misses the important rheostat positions. Rheostat positions can also be obscured when SSM data are analyzed as the average/median for each position (*e.g.*, (28, 29), see (30) for further discussion).

Finally, examination of the RheoScale scores for the various proteins described below suggests the neutral, rheostat, and toggle substitution behaviors themselves fall along a continuum: Studies have observed (i) neutral positions (11, 31, 32); (ii) positions for which substitution outcomes are *not* neutral but do not meet the rheostat score threshold, which have been referred to as “non-neutral” and “modest” rheostat positions in various publications (33, 34, 35); (iii) rheostat positions with substitution outcomes that sample the entire range (11, 33, 36); (iv) rheostat positions for which many substitutions are deleterious or catastrophic, *i.e.*, at least half of the range is sampled, but the sampling is skewed towards the most deleterious outcomes (32, 37); and (v) toggle positions (1, 17).

## Rheostat positions are widespread and biologically important

### Rheostat positions are present in many protein types

The continuum of neutral, rheostat, and toggle substitution behaviors described above were collated from a wide variety of protein types: Since the first identification of rheostat positions was limited to homologs in the LacI/GalR family (11), the next generation of studies was designed to determine how widespread rheostat positions might be. To simplify the vast possibilities, one approach was to sample proteins that evolved under divergent structural constraints: globular soluble proteins, integral membrane proteins, and intrinsically disordered proteins (Table 1). Rheostat positions were identified in all three structural types; definitions for their various functional parameters measured and technical considerations important to these studies are listed in Table 1 footnotes. In addition, retrospective analyses of other SSM datasets assessed with high-throughput techniques identified rheostat positions in a variety of other proteins (*e.g.*, (29, 38, 39, 40, 41, 42, 43)). Rheostat positions can also be retrospectively inferred from alanine scans: Substitutions with partially detrimental (*i.e.*, not catastrophic) or enhancing functional outcomes are likely to the rheostat positions (44). Our review of these ever-expanding datasets has not been exhaustive and, given the growing popularity of these high-throughput approaches, we expect many more rheostat positions will be identified in a wide variety of protein structures.

**Table 1:** Model proteins used in studies of rheostat positions

Protein (abbreviation; # of amino acids per monomer)^a^	Overall function	Parameters measured^b^^,^^c^	# Rheostat positions identified to date^d^	Citations
Globular soluble				
*E. coli* lactose repressor protein (LacI; 360 aa)	Allosterically regulated transcription repressor	1. *In vivo* repression benchmarked against DNA binding affinity 2. *In vivo* induction at high IPTG^e^ concentration; EC_50_ for *in vivo* IPTG induction 3. IPTG binding affinity 4. Allosteric response	1. 9 of 12 positions *via* semiSM and assayed by high-resolution *in vivo* repression assays; 1 of 1 position verified by biochemical assays. 2. 149 of 320 positions *via* semiSM assessed by low-resolution *in vivo* assays	(1, 3, 4, 11, 17, 37, 54, 57, 60, 139),^f^
Engineered *E. coli* LacI/GalR paralogs (LLhX series, ∼320 aa)	Allosterically regulated transcription repressors	1. *In vivo* repression benchmarked against DNA binding affinity	65 of 112 positions *via* semiSM	(5, 6, 7, 8, 10, 11, 12, 17, 49)
Human liver pyruvate kinase (hLPYK; 543 aa)	Allosterically regulated enzyme	1. Apparent^g^ binding affinity for substrate (K_app-PEP_^h^) 2. Apparent binding affinity for allosteric activator (K_FBP_^h^) and allosteric inhibitor (K_Ala_^h^) 3. Allosteric coupling between active site and each of the allosteric sites (Q_FBP_, Q_Ala_).	1. 18 of 33 positions *via* semiSM substitutions 2. >160 (of 543) rheostat positions estimated from alanine substitutions	(17, 33, 36, 44, 59, 99, 100),^i^
*Zymomonas mobilis* pyruvate kinase (ZmPYK; 475 aa)	Non-allosterically regulated enzyme	Apparent^g^ binding affinity for substrate (K_app-PEP_)	Zero of 26 positions *via* semiSM	(34)
Human aldolase A (aldolase; 364 aa)	Non-allosterically regulated enzyme	Apparent^g^ binding affinity for substrate (K_app_), cooperativity, structures	1 of 1 position *via* semiSM	(106)
Integral membrane				
Sodium taurocholate co-transport-ing polypeptide (NTCP; 349 aa)	Bile acid and statin transport	Transport at a fixed substrate concentration normalized to cell surface expression, Michaelis constant (K_m_), maximal velocity (V_max_), computed energetic effects on structure	4 of 4 positions *via* SSM	(32, 50, 55)
Organic anion transporting polypeptide 1B1 (OATP1b1; 691 aa)	Hepatic drug transport	Transport at a fixed substrate concentration normalized to cell surface expression	1 of 1 position *via* semiSM; 11 estimated from cysteine-scanning variants	(140)
Intrinsically disordered				
Human MHC class II transactivator protein (CIITA; isoform 3; 1130 aa)	Eukaryotic transcription-al activator	*In vivo* transcriptional activation normalized to cellular concentration	4 of 7 positions *via* semiSM	(35)

a One criterion in selecting these proteins was that all functional assays were either independent of or accounted for changes protein stability/cellular concentration. For example, in catalytic reactions, enzyme concentration is extremely low and measurements of K_d,app_ and allosteric coupling are independent of concentration. Despite these efforts, substitution effects could not be disentangled for some “dead” variants, which derive from either lost function or structural disruption (33, 34, 36). Fortunately, at rheostat positions, dead variants comprise the minority of substitutions and thus do not impact conclusions about the overall roles of these positions.

b Functional assays should allow assessments for dozens, if not hundreds, of amino acid substitutions. For enzymes, the ability to carry out assays after partial purification increased throughput (31, 33, 34, 59, 106). For other proteins, protein purification is precluded with *in vivo* measurements, with the caveat that measured changes can arise from changes in multiple biochemical parameters, including effects on protein concentration/stability. Fortunately, the latter can be detected and compensated using parallel experiments to measure concentration (*e.g.*, (11, 35, 50, 55, 141)). The possibility of combined outcomes is especially important to keep in mind for “deep mutational scanning” studies (*e.g.*, (38, 41, 48, 142)), which – in addition to combining potential changes from multiple biochemical parameters – often make assumptions to connect the measured outcome (variant frequency within a genetic population) to a substitution’s effect(s) on the protein of interest; many of these studies are now adopting parallel assays of protein concentration (*e.g.*, (143, 144, 145)).

c To detect the intermediate functional outcomes common at rheostat positions, assay resolution is critical. With a few exceptions, biochemical and biophysical studies usually have smaller experimental errors than high-throughput *in vivo* studies. Experimental errors should be especially considered for assays with a narrow overall range; multiple biological and technical replicates for wild-type protein (10 or more) can provide a good description of error (31, 33, 35, 106). Fortunately, high-throughput robotics are being developed that allow biochemical measures for hundreds of variants (45, 146, 147) and will greatly advance experimental studies of rheostat positions.

d The first number in this column (*e.g.*, 9 or 149 for LacI in row 1) gives the number of rheostat positions experimentally identified by the method indicated; the second number (*e.g.*, 12 or 320 for LacI) gives the total number of positions experimentally tested in the study.

e Inducer “IPTG” is isopropyl β-d-1-thiogalactopyranoside.

f The AlloRep database (54, 139) collates the published substitution outcomes known for LacI. For both LacI and its engineered paralogs, *in vivo* repression and induction assays have been extensively benchmarked against *in vitro* biochemical measurements and direct measures of *in vivo* concentration ((4, 5, 6, 7, 8, 10, 11, 12, 49, 58, 148) and many other LacI studies in (54, 139)).

g Apparent binding affinities and allosteric coupling were derived from analyses of kinetic assays; mathematical descriptions for these parameters are in (59, 101, 102, 149).

h Substrate “PEP” is phosphoenolpyruvate; allosteric activator “FBP” is fructose-1,6-bisphosphate; allosteric inhibitor “Ala” is free alanine.

i The PYK-SubstitutionOME database (44) collates the published substitution outcomes known for many pyruvate kinase homologs.

Several surprising outcomes were noted in these studies. First, despite their widespread occurrence in many protein types, rheostat positions for function have not yet been found in some globular proteins (34, 45); this is discussed further below. A second surprise was the identification of rheostat positions in an intrinsically disordered (ID) region that comprises the transcriptional activation domain of the eukaryotic transcription factor CIITA (35). Originally, the CIITA ID region was anticipated to contain either (i) neutral positions, as was the case for the intrinsically disordered activation domains of p53 (22) and PPARγ (46) or (ii) to only be affected by substitutions that altered the balance of acidic and hydrophobic residues, as has been proposed for other ID activation domains (26, 47). Instead, the CIITA study shows that, like globular proteins, functional sensitivity to single substitutions and the presence/absence of rheostat positions cannot be generalized among ID regions. A 2023 survey of the MAVE database (48) shows that ID regions are seldom targeted with SSM or semiSM experimental designs, although those designs are used to interrogate the globular domains present in the same proteins. Thus, more SSM/semiSM studies are needed to understand the sequence/function relationships ID regions.

### Substitutions at rheostat positions have a biological impact

The existence of rheostat positions is interesting from a purely academic perspective, but their significance hinges on whether changes at these positions have measurable biological impacts. For known rheostat positions, there is both direct and indirect evidence of biological importance: In the LacI/GalR homologs, any measurable change in repression (≥2-fold) was sufficient to alter bacterial growth (49). In human NTCP, the site of a medically-relevant polymorphism was a rheostat position; furthermore, the functional difference between the two naturally occurring variants was substrate-dependent (50). In hLPYK, the locations of some rheostat positions coincide with known disease-causing mutations (30). In the database of clinically relevant mutations in von Willebrand factor, different amino acid substitutions at the same position are associated with different disease severity (51), consistent with the features of rheostat positions; likewise, biophysical assessments of von Willebrand factor show that some pathogenic variants cause partial loss of function whereas others lead to gain of function (52).

These studies suggest that non-catastrophic effects on protein function can have biological consequences. Different levels of functional change might lead to a range of disease severities, or disease might result when variant function crosses a biologically critical threshold (*e.g.*, (23)). Note that the disease threshold will differ for each protein, and even for a single protein under different environments or epistatic conditions (*i.e.*, other proteins in the organisms have amino acid changes) (reviewed in (53)). Furthermore, in some proteins, the most deleterious substitutions may cause fetal death, so that only the intermediate range of reduced function are seen as disease-causing. Nonetheless, any correlation of disease with mutations at rheostat positions is strong evidence for biological impact. Thus, as noted by Miller *et al.* (1), one goal of computational predictions should be to predict intermediate functional outcomes rather than imposing binary assignments such as “benign/detrimental”. Indeed, such intermediate functional outcomes might be one explanation for the 11% of “uncertain” human missense mutations with intermediate pathogenicity scores by the recently published predictor “AlphaMissense;” (this study only reported confidence in substitutions predicted to be either “likely benign” or “likely pathogenic” (19)).

It is also interesting to think about the biological consequences of substitutions that lead to gain-of-function. As noted above, such gain-of-function outcomes were observed for ∼10% of substitutions in semiSM studies (*e.g.*, (11, 33, 35, 50, 54, 55)). This suggests that the wild-type proteins have *not* evolved to their maximum possible function. One reason for this could be that “enhanced” function is biologically detrimental (*e.g.*, (56)). Another reason could be that the protein evolved to be “good enough”; there was no pressure to evolve functional perfection. Yet a third reason is that enhancing one aspect of function may simultaneously alter another; the related functions must simultaneously evolve to be “good enough.” Future studies of gain-of-function substitutions will advance the engineering of protein reagents for biotechnology.

### Rheostat positions can be prevalent within individual proteins

**Figure 3:**
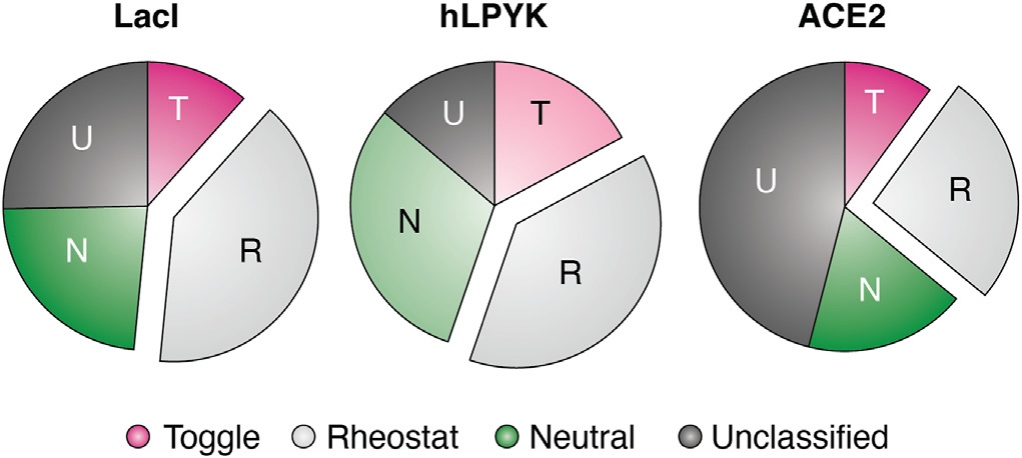
**Prevalence of position classes in representative proteins.** The fraction of toggle positions (“T”) is shown with *magenta*; the fraction of rheostat positions (“R”) is shown with *light gray*; and the fraction of neutral positions (“N”) is shown with *green*. The fraction of positions for which no assignment could be made are shown in *dark gray* (unclassified, “U”); at these positions, data were either too sparse or their RheoScale scores did not meet any threshold (most often, such positions were non-neutral “moderate” rheostat positions (33)). For LacI, the numbers of rheostat, toggle, and neutral positions (33) were estimated from low-resolution *in vivo* repression assays (57, 58); as detailed in (33), the number of neutral positions is likely an over-estimate and the number of rheostat positions is likely an underestimate. For hLPYK, numbers were estimated from a whole-protein alanine scan (44, 59); the number of rheostat positions (based on the number of alanines with either gain- or partial-loss-of-function) is a low estimate; positions counted in the neutral and toggle categories are likely high over-estimates (*lighter colors*). For ACE2, variants were generated by SSM of the ACE spike binding interface, and functional outcomes were assessed *via* binding surface-expressed ACE2 to soluble, GFP-tagged SARS Cov2 spike protein and measured with flow cytometry (29, 40).

Demonstrating the importance of rheostat positions also requires assessing their prevalence within individual proteins. In LacI, retrospective analyses of the Miller lab’s semiSM studies (57, 58) suggest that at least 40% of this protein’s positions are rheostat positions ((33); Fig. 3). In hLPYK, results from a whole-protein alanine scan (59) indicate that >30% of this protein’s positions are rheostat positions ((44); Fig. 3). In the region of human ACE2 that binds the SARS CoV2 spike protein, 26% of the 117 positions assessed were rheostat positions for spike binding ((30); data from (29) were later published in (40)). With these high numbers in three distinct proteins, it is safe to conclude that rheostat positions can be prevalent within individual proteins!

Nonetheless, rheostat positions have *not* been identified in some proteins. No rheostat positions were observed in ZmPYK when substrate affinity was assessed at 26 positions (34). Instead, the variants at most positions could be categorized as either “near wild-type” or “dead.” At first glance, many of these ZmPYK positions appeared to exhibit classic toggle behavior; however, they failed to meet two criteria: (i) Only about half of the variants for each position lacked activity, in contrast to toggle positions, for which most variants lack activity. (ii) Amino acid substitutions that retained near wild-type function showed few chemical similarities. The ZmPYK data were also striking in comparison to its homolog hLPYK, which is estimated to have >30% rheostat positions (Fig. 3). A second example of paltry substitution effects on a functional parameter was found in studies of the alkaline phosphatase PafA. In comprehensive valine/glycine/alanine scan of this protein’s enzymatic function, only 17 of its >520 substituted positions exhibited non-catastrophic, non-neutral effects on the Michaelis constant K_m_ (45).

Together, these results suggest that some proteins have functions that are not substantially impacted by single amino acid substitutions. The evolution of such proteins must instead be accomplished by combinations of substitutions at multiple positions, which can produce change *via* (i) additive combinations of small effects (*e.g.*, 2- to 3-fold) (60, 61, 62) and/or (ii) larger, non-additive effects arising from epistasis among substitutions with small/neutral effects (63, 64, 65, 66, 67)). By extrapolation, these proteins may lack rheostat positions for any of their functional parameters. This difference is not related to either (i) the assayed function – both hLPYK (with many rheostat positions) and ZmPYK (with no identified rheostat positions) catalyze the same enzymatic reaction (reviewed in (44)), or (ii) overall protein fold – hLPYK (*e.g.*, (68)) and ZmPYK (69) have similar monomeric structures. In the future, it will be important to determine which biophysical properties allow the presence of rheostat positions in some proteins and not in others.

## Characterization of rheostat positions reveals complex relationships

### Both rheostat and neutral positions can be “nonconserved”

Another historical reason why rheostat positions were overlooked is that many have assumed that nonconserved positions are neutral and did not include them in experiments. Thus, it is important to differentiate these two types of nonconserved positions and to directly test the association between nonconservation and functional neutrality. To that end, one avenue of study has been to identify whether any evolutionary features found in MSAs can discriminate neutral and rheostat positions.

When analyzing MSAs, one important insight is recognizing that “nonconservation” can be defined many ways. First, “nonconserved” is contextual. One context is provided by the sequence identity threshold used to define a protein family (53). For example, rheostat positions in the LacI/GalR family are nonconserved in the super-family and often conserved within subfamilies (9, 11). A second context is provided by the taxonomic distribution of the protein family. For example, two protein families with correlations between evolutionary features and position types—LacI/GalR and pyruvate kinase (PYK)—are taxonomically diverse (9, 34, 70). LacI/GalR homologs are present in most bacteria; LacI/GalR paralogs bind different DNA and allosteric ligands (9, 71). PYK homologs are present in all domains of life; isozymes catalyze the same chemical reaction with varied allosteric regulators (34, 70, 72, 73, 74). In contrast, the CIITA family is limited to vertebrates (75, 76), and no correlation was observed between evolutionary features and rheostat positions in human CIITA (35). Even though only a few positions were tested in CIITA, it seems reasonable to infer that MSA analyses are of limited utility for taxonomically restricted proteins.

Second, within an MSA, several types of “nonconservation” can occur: Changes at some pairs (or larger clusters) of positions are correlated, suggesting that they co-evolve (*e.g.*, (77, 78, 79, 80, 81, 82, 83, 84, 85, 86, 87, 88, 89, 90, 91, 92)). At other positions, amino acid changes correlate with the branch points of the MSA’s phylogenetic tree (*e.g.*, (93, 94, 95, 96)). When compared to experiments, all of these patterns can identify “important” protein positions (*e.g.*, (9, 77, 79)), and all patterns appear to reflect various biological or biochemical pressures that occurred during protein evolution. Thus, Martin *et al.* (31) hypothesized that positions with the *least evidence* of a changing pattern (*i.e.*, most random evolutionary change quantified by a “least-patterned” score) would be likely candidates for neutral positions.

**Figure 4:**
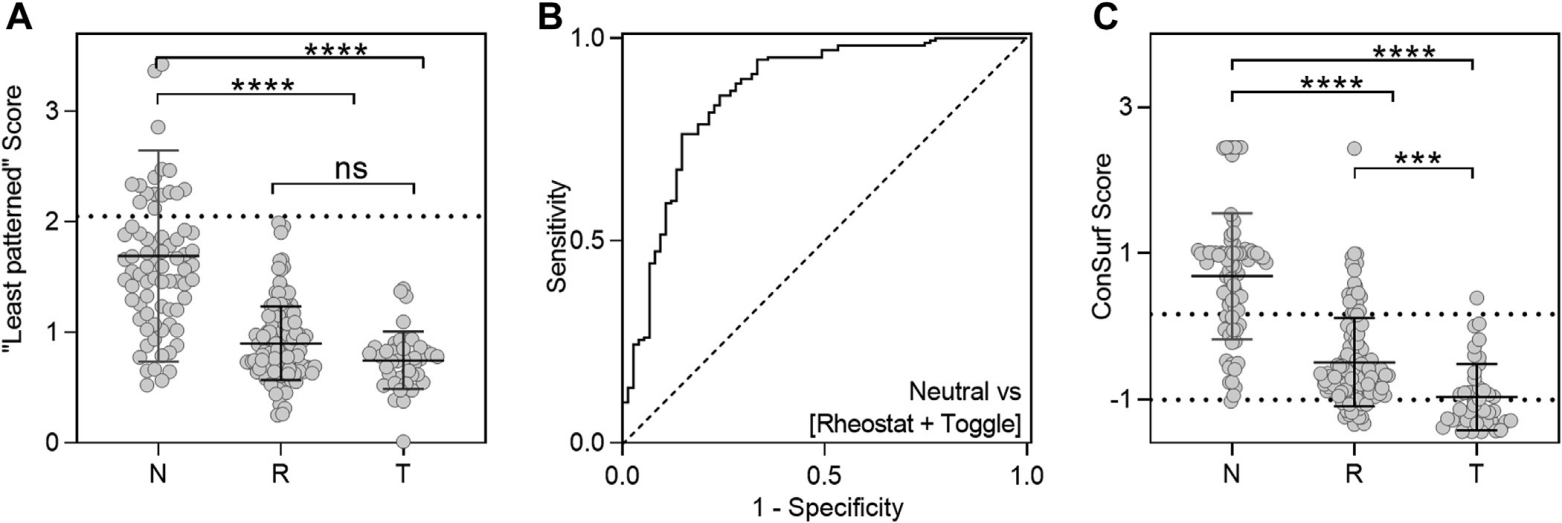
**Evolutionary signatures of neutral (N), rheostat (R), and toggle (T) positions in LacI.** For each position in LacI, an overall substitution outcome was determined using a semiSM set of variants assessed with *in vivo* functional assays (33, 58); for the resulting groups of N, R, and T positions, scores derived from various MSA analyses were compared. *A*, the “least patterned” score reported in (31) distinguishes neutral positions from rheostat/toggle positions; the dashed horizontal line illustrates a threshold that perfectly discriminates a subset of the neutral positions. *B*, the receiver–operator curve (ROC) for the true positive (sensitivity) and false positive (1 − specificity) outcomes that arise from using all possible thresholds of the least-patterned score to discriminate the set of neutral positions *versus* the set of [Rheostat + Toggle] positions; the area under the curve (AUC) is 0.8669 with a 95% confidence interval of 0.8128 to 0.9210; the curve for a perfect predictor would be a step function with AUC 1.0; the *dashed line* represents a useless predictor with AUC 0.5. *C*, distributions of LacI ConSurf raw scores for the three positions classes (33). ConSurf was also successful at discriminating neutral and non-neutral [Rheostat + Toggle] positions, with the AUC of a ROC curve of 0.8369 (See Supplemental Fig. S8 in (33)). The *dashed lines* show the statistically-determined thresholds for separating these three categories (33). Given the substantial additional effort required to calculate the “least patterned” score of (31), ConSurf is a practical choice. For (*A*) and (*C*), ANOVA was carried out using Tukey’s multiple comparisons test of the means; ∗∗∗∗*p* < 0.0001; ∗∗∗*p* = 0.0003; “ns”, not significant.

When the least-patterned hypothesis was tested experimentally in hLPYK, results were encouraging (31). Although it is impossible to prove a negative (as extensively discussed (31)), several of the substituted positions showed zero change in the functions monitored, and others showed only modest deviations from wild-type. A second set of neutral positions was gleaned from a high-throughput/low-resolution study of LacI (33, 57, 58). When the least-patterned scores of (31) were calculated for LacI, a subset of the neutral positions was highly discriminated from rheostat and toggle positions (Fig. 4, *A* and *B*). Since neutral positions provide a much-needed control for understanding the biophysical underpinnings of rheostat positions, future studies should be geared towards identifying neutral positions in more types of proteins.

### Evolutionary signatures have some utility for discriminating rheostat, neutral, and toggle positions

Given the success of combining MSA analyses to identify a subset of neutral positions, the question was raised whether a similar strategy would effectively discriminate the three classes of rheostat, toggle, and neutral positions. To that end, the LacI experimental dataset was compared against results from 12 independent MSA analyses, as well as combinations of analyses. Each analysis suggested differences among the three classes, but the three distributions of scores showed substantial overlap (33). Of these, the phylogenetic analysis ConSurf best distinguished the three classes ((33); Fig. 4*C*).

ConSurf uses a tripartite function to quantify conservation at each position (MSA column); this information is then combined with information from the protein’s phylogenetic tree to generate a score (94) that can be used to discriminate positions with whole-family conservation, subfamily conservation, and nonconservation (*e.g.*, (9, 33)). The set of positions that show subfamily-specific conservation are enriched for rheostat positions (33). Conveniently, ConSurf is available as a recently updated web server and as a stand-alone pipeline ((94); https://consurf.tau.ac.il). Notably, ConSurf separated LacI rheostat and toggle positions much better than co-evolutionary methods, and a combinatorial approach showed no improvement. When phylogenetic scores were used to predict the locations of rheostat positions in hLPYK, experiments showed that several of these positions were highly rheostatic for multiple functional parameters; none of the predicted positions were neutral (33).

Despite this success, the distributions of the scores for neutral, rheostat, and toggle positions show significant overlap. One reason for this overlap could be that the following assumptions inherent to MSA analyses are not always appropriate:

#### Assumption 1

Every MSA analysis implicitly assumes that the role of each position (MSA column) is the same for all homologs in the family. This need not be so. For example, when subfamilies of the LacI/GalR family were separately analyzed, some conserved and co-evolving positions were in different locations on their common structural architecture, suggesting that these positions play roles unique to each subfamily (79). Furthermore, when analogous positions were experimentally assessed in ten engineered LacI/GalR paralogs, their substitution classes were not always the same. For example, several positions showed rheostatic substitution behavior in 80% of the homologs tested but exhibited different behaviors (*i.e.*, toggle or neutral) in the remaining homologs (11, 17). It would not be surprising to find that positional contributions to function also vary among natural paralogs.

#### Assumption 2

A second assumption that may not always be true – and therefore may contribute to the observed overlap in Figure 4 – is implicit in MSA analyses: predicting substitution classes from MSAs assumes that all homologs in the family actually *have* rheostat positions and that the protein function can be tuned by single substitutions. As described above, available data for ZmPYK suggests that this is *not* true for all PYK homologs (34).

#### Assumption 3

MSA analyses detect amino acid conservation or patterns of change that are assumed to arise from evolutionary pressures on the protein. However, every protein experiences multiple pressures to maintain various structural and functional features (described in (34, 97)). Since the various pressures all contribute to the patterns of change observed in MSAs, this likely confounds assigning MSAs signatures to any one functional parameter or substitution behavior (*e.g.*, functional rheostat positions).

Since MSAs are central inputs to essentially all algorithms that predict substitution outcomes, these three assumptions must be kept in mind because their associated limitations may limit the amount and types of information that can be extracted from sequence alignments. When these assumptions are not true, one result might be the overlap exemplified in Figure 4. Nonetheless, as detailed above, MSA-based predictions of rheostat and neutral positions can be useful for guiding experiments and selecting subsets of positions that are enriched for the desired behavior.

### Multiple aspects of function can be simultaneously altered by substitutions at rheostat positions

Since patterns of change observed in MSAs can reflect multiple evolutionary pressures, one might expect substitutions at rheostat positions to simultaneously affect multiple functional parameters. Indeed, using high-resolution biochemical assays, many examples of this behavior have been found.

For example, substitutions at one LacI rheostat position simultaneously modulated DNA binding, effector binding, and the magnitude of allosteric response (4). Even in low-resolution *in vivo* assays, at least 27 LacI rheostat positions had simultaneous effects on repression and induction (98). In a second example, a rheostat position in the bile acid/statin transport protein NTCP showed ligand-dependent effects on each substitution’s outcome (50); this indicates that the substitutions altered substrate specificity (*i.e.*, which substrate is the most preferred ligand; further discussed in (12)). Furthermore, changes were observed in both kinetic parameters (K_m_, V_max_), with uncorrelated outcomes (50, 55). In a third example, experiments for hLPYK were designed to simultaneously assess three apparent binding affinities and two allosteric couple constants (Table 1). Substitutions at hLPYK rheostat positions often affected more than one parameter, with at least one position affecting all five; other hLPYK positions showed rheostat outcomes for some parameters and toggle outcomes for others (17, 33, 36, 99, 100).

**Figure 5:**
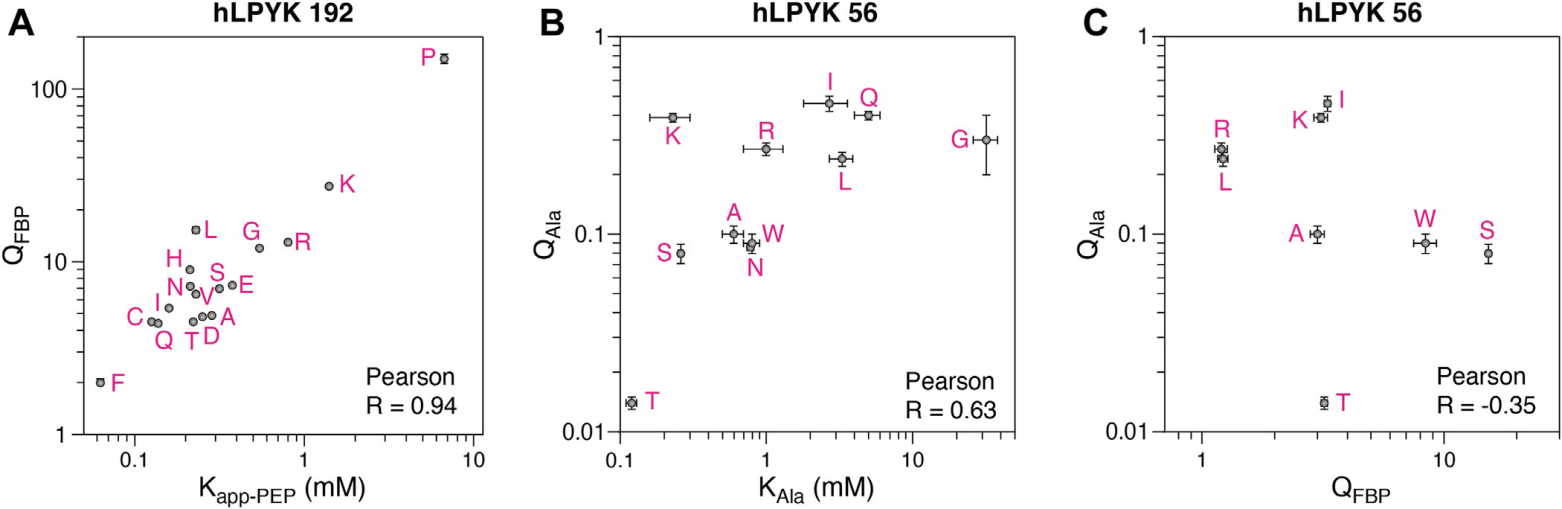
**Correlations of measured parameters for substitutions attwohLPYK rheostat positions.** The enzymatic reaction and allosteric regulation of this enzyme are described in Table 1. Functional parameters measured for each of the variants at hLPYK (*A*) position 192 and (*B* and *C*) position 56 are shown with *dots*; error bars are errors of fit and may be smaller than the symbol size. Data and analyses were taken from (33, 36, 100). For position 56, in addition to substitutions shown on the plots, S56C had no measurable activity; S56N and S56Q showed no binding for the allosteric activator FBP; S56G showed so little binding for FBP that QFBP could not be measured. The allosteric coupling constants (Q) are derived from a ratio of binding affinities and are unitless. This figure also provides an example of which physico-chemically similar amino acid side chains exhibit different outcomes (*e.g.*, at position 56, S and T differ for Q_Ala_ and Q_FBP_, and N and Q have disparate K_Ala_).

For parameters related to allosteric regulation, simultaneous effects are expected: Allosteric coupling is mathematically defined as a non-unity ratio of substrate binding in the absence of effector *versus* substrate binding in the presence of saturating concentrations of the effector (*e.g.*, (59, 101, 102, 103)). As such, simultaneous effects on binding and allosteric coupling arise from the same functional change (*e.g.*, Fig. 5*A*). However, deviations from perfect correlation (*e.g.*, Fig. 5*B*) and uncorrelated effects on other parameters (*e.g.*, Fig. 5*C*) have also been observed. Since the functions in this hLPYK example involve three separate binding sites, each separated from the others by ≥20 Å, the combined results suggest that substitutions at rheostat positions have long-range effects that are mediated by changes in the protein structure.

## Structural features that might enable functional rheostat positions

### Functional rheostat positions have no obvious relationship to binding sites

**Figure 6:**
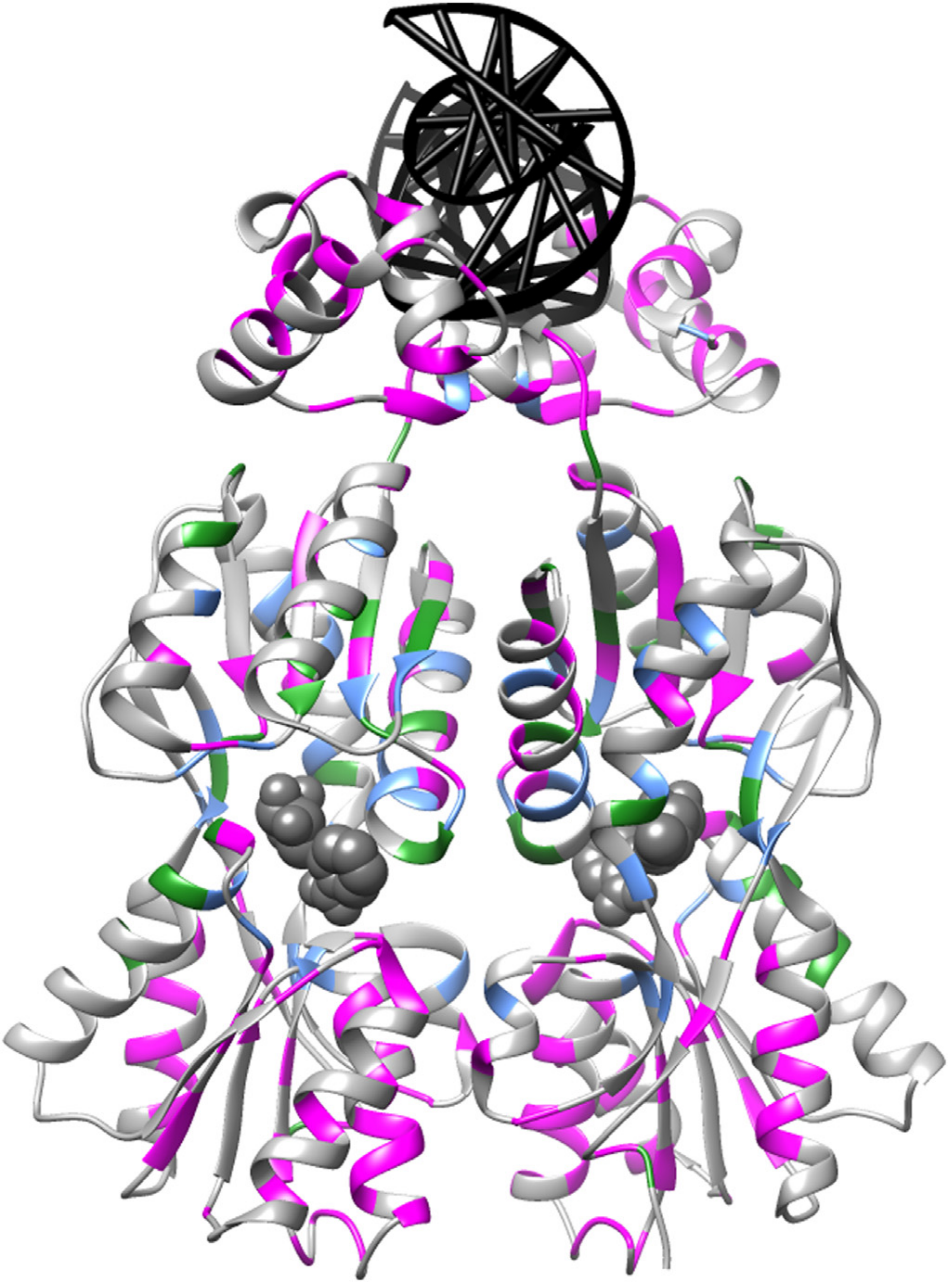
**Locations of rheostat positions on the LacI homodimer.** For all positions in LacI, the Miller lab used semiSM and low-resolution assays (57, 58) to assess each variant for effects on (i) transcription repression—which is mediated by DNA (*black ladder*) binding and (ii) transcription induction—which is mediated by binding an allosteric inhibitor such as IPTG that diminishes DNA binding (binding sites denoted with ligand in *gray spheres*). *In vivo* data have been extensively benchmarked against biochemical analyses of purified proteins (summarized in (54)) and were analyzed to identify rheostat positions (33). Rheostat positions that alter either repression (*magenta*) or induction (*green*) are shown on pdb 1EFA (104). Positions that were rheostatic for *both* functional outcomes are in *blue*; such “multiplex” rheostat positions were also identified in pyruvate kinase (17, 36). Notably, the side chains for many of the LacI rheostat positions are solvent-exposed. Forty percent of LacI positions had rheostatic behaviors, which exceeds the numbers of either toggle or neutral positions (33). This illustration was created in UCSF Chimera (137).

To understand the mechanisms that give rise to the noncanonical substitution outcomes at rheostat positions, an obvious line of inquiry is to determine their locations on protein structures. A simple explanation could be that a position’s substitution behavior is related to its proximity to ligand binding/active sites: Toggle positions might directly contact the ligand; rheostat positions might occur in the “second shell” from the ligand; functionally neutral positions might be located far from the ligand binding site, including on the protein’s surface. However, to date, no such structural pattern has been observed for the locations of functional rheostat positions. The side chains of some rheostat positions directly contact ligands; many others do not. Visual inspection shows that the locations of rheostat positions are scattered all over the structures of LacI (*e.g.*, Fig. 6) and hLPYK (33). In contrast, many ZmPYK positions close to its active site were not rheostat positions (34).

Thus, the substitution effects of many rheostat positions must propagate “long range” through the structure to some region of the protein that is energetically coupled to a binding/active site. In other words, altered ligand binding can arise even if there are no observed changes in the binding site when the ligand is absent. Future studies should identify the biophysical properties that allow such propagation in some proteins and not others. Such studies could provide key insights into which types of proteins can contain rheostat positions.

### Functional rheostat positions do not cause substantial conformational change

Another question about substitutions at rheostat positions involves assessing what changes occur in the proteins’ folded conformations. For a protein’s overall secondary/tertiary/quaternary structure, various studies have provided both indirect and direct evidence that suggests the answer is “Very little.”

The indirect evidence is that most substitutions at rheostat positions *have* measurable functions. This would not be possible if the substitution substantially distorted the protein’s overall structure. Indeed, all substitutions at rheostat positions in LacI and engineered LacI/GalR paralogs were capable of binding DNA, even if their binding affinity was too weak to repress transcription (11). Likewise, most substitutions at hLPYK were able to catalyze the native reaction and bind the same allosteric effectors (44); this would not be possible if the conformation was substantially changed. Even glycine and proline substitutions at rheostat positions, which are expected to cause drastic structural changes (albeit from historical studies of toggle positions), allowed measurable function in several model proteins (4, 33). In several instances, proline was not even the worst-performing variant.

Comparison of paralog structures also suggests no global conformational changes. For example, the whole protein C^α^ RMSD for LacI (PDB 1EFA; (104)) and *Escherichia coli* PurR (PDB 1WET; (105)) is <1.5 Å; nonetheless, the intra-protein interactions of the nonconserved rheostat positions show substantial local re-packing, with different amino acids at analogous positions contacting different partners (3). This type of side-chain re-packing (*i.e.*, plasticity) was also computationally predicted for NTCP (55). When substitutions were modeled for the highly buried side chain at rheostat position 271, only localized re-packing was observed; essentially no changes were predicted for the overall conformation in either the open or the closed states. Another 12 position NTCP positions were predicted to have similar structural plasticities. Finally, crystal structures of substitutions at a rheostat position in human aldolase A showed essentially no change beyond the immediate region of the substituted side chain (106). Thus, the protein regions that accommodate rheostat positions and their substitutions appear to show structural plasticity.

However, none of the studies described above rule out altering the distributions of alternative conformations on each protein’s conformational landscape. Individual substitutions—even at the same position—could have unique effects on this landscape. Indeed, a conclusion from structural studies of human aldolase A is that substituting a rheostat position shifts its conformational ensemble, with some of the alternative conformations being inactive (106): Four variants at a single rheostat position crystallized in the same space group and their structures had the same fold; nevertheless, the different resolutions of the four variants correlated with a changing functional parameter. A fifth variant at the same position crystallized but did not diffract, had unchanged secondary structure by circular dichroism (suggesting that its overall fold was intact), yet lacked detectable enzymatic activity. Thus, the authors proposed that substitutions at this functional rheostat position shifted the aldolase conformation (106), similar to the shifts observed with fluorescence spectroscopy for substitutions that altered its substrate specificity (107). A second example of a protein for which enzymatically inactive substitutions arise from shifts in the conformational ensemble is the alkaline phosphatase PafA (45).

### Substitutions at functional rheostat positions might cause functionally important changes in dynamics

**Figure 7:**
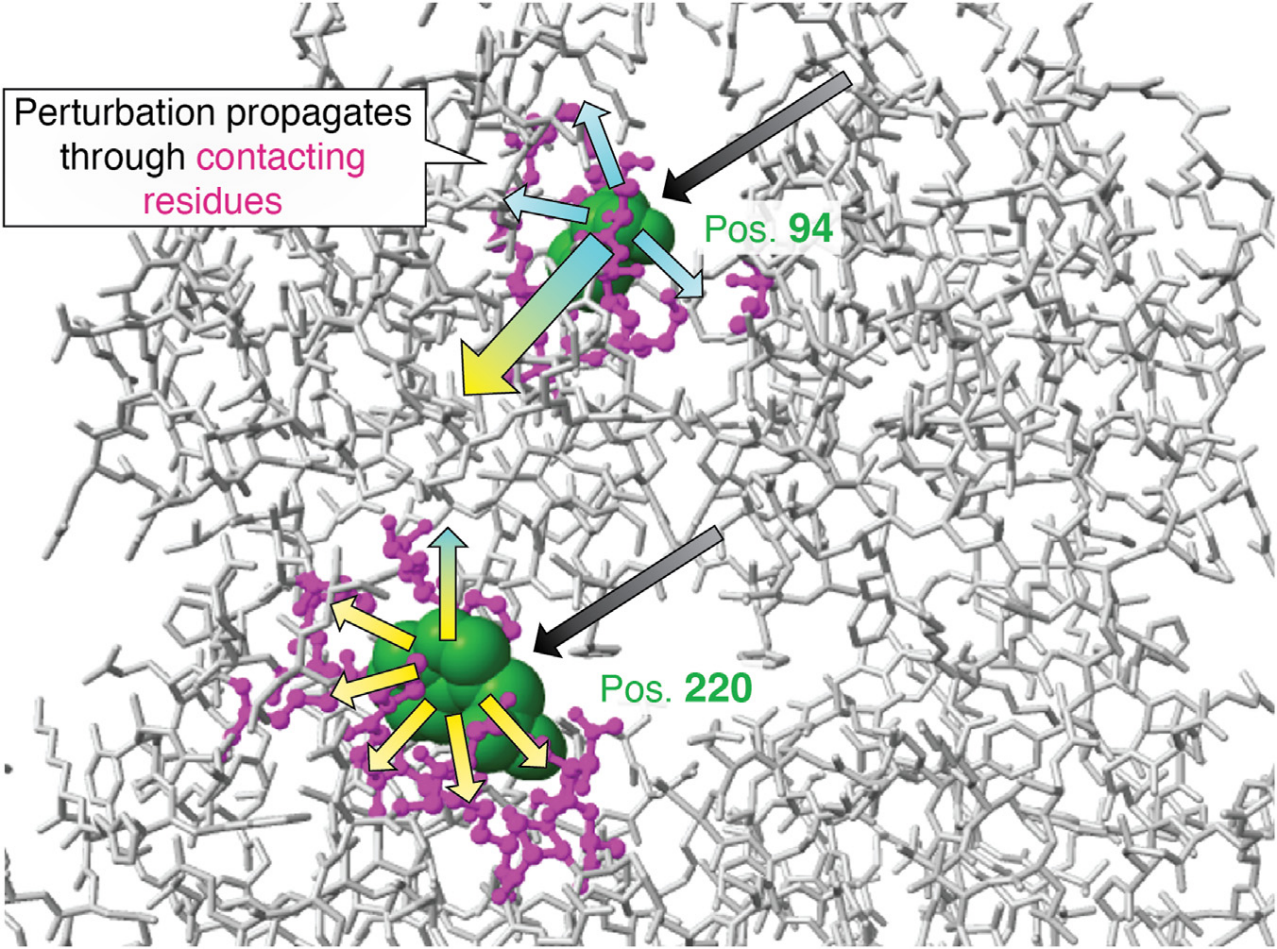
**Protein structures are anisotropic networks.** In an anisotropic network, the propagation of a perturbation from node 1 to node 2 differs from the propagation from node 2 to node 1 (*e.g.*, (37)). One consequence of anisotropy is that coupled positions can have unique substitution outcomes. In the example illustrated here, a perturbation (*upper black arrow*) at position 94 (*green space filling*) propagates through contacting residues (*magenta ball and sticks*) to the rest of the protein structure (*cyan arrows*), including to position 220 (*large shaded arrow*). Because position 220 has different contact partners than position 94, the same size perturbation (*lower black arrow*) propagates differently through the structure (*yellow arrows*), and dynamics coupling from position 220 to position 94 differs (*smaller shaded arrow*). This illustration uses the LacI pdb 1EFA (104) and was created with ChimeraX (138).

Another possible mechanism by which substitutions at rheostat positions could exert their long-range, through-structure effects on protein function is by altering protein dynamics. Support for this hypothesis can be found in the dynamics computations for variants at a LacI rheostat position (37). In this study, the protein conformation was subjected to all-atom molecular dynamics simulations and then treated like a 3D network of coupled balls (atoms) and springs (bonds). In this model, when one atom is perturbed—by Brownian motion or ligand binding—vibrations propagate through the anisotropic network of the 3D structure (*e.g.*, Fig. 7); some of these vibrations reach the atoms in the binding site. When an amino acid is substituted with another side chain, the resulting alterations effectively “re-wire” that local section of the network. Although the resulting conformational changes look small and localized, effects on vibrational propagations can be large. Intriguingly, for substitutions at a LacI rheostat position, changes in the flexibilities of the DNA binding contacts correlated with changes in the measured DNA binding affinities (37).

As such, the LacI dynamics study suggests a potential approach for predicting substitutions with partially deleterious or enhancing outcomes on function. In support of this approach, other computational studies have correlated such dynamic changes with pathogenic missense substitutions (108) and leveraged this information to design enhancing substitutions (109). Amino acid-dependent, long-range coupling has also been observed experimentally *via* NMR (110, 111). In future studies, such dynamic correlations should be tested in other proteins and for a range of ligand types.

### Substitutions at functional rheostat positions have unknown effects on stability

To date, most studies of rheostat positions have focused on changes in protein function, using assays that were largely insensitive to changes in protein stability (the equilibrium between folded and unfolded protein, measured by the free energy of unfolding, ΔG_u_). However, given the large numbers of amino acid substitutions that are known to alter ΔG_u_ (*e.g.*, (21, 112, 113, 114, 115, 116, 117)), the relationship between function and stability should also be considered for substitutions at rheostat positions.

One possibility is that ΔG_u_ must substantially favor the folded state to observe the functional modulation associated with destabilizing substitutions. In support of this hypothesis, several studies have concluded that mutations that increase protein stability enable natural and directed evolution of function variation (*e.g.*, (118, 119, 120, 121)) and that ancestral proteins (prior to the evolution of functional variation) might have had higher stability (122). High stability is known for at least one of the model proteins with a high prevalence of functional rheostat positions: tetrameric LacI. With a ΔG_u_ of −60 kcal/mol for the wild-type protein (123), most single mutations in LacI probably do not alter stability sufficiently to cause a biochemically or biologically meaningful effect.

Related to this idea, since the stabilities of smaller proteins have been shown to be more sensitive to perturbations than larger proteins (112, 124), the presence (or prevalence) of functional rheostat positions may depend upon protein size: In smaller proteins, substitutions that rheostatically alter function might have a greater likelihood of producing a concomitant, catastrophic effect on stability. In larger proteins, substitutions that alter function might have a greater chance that they can be accommodated without causing catastrophic changes in ΔG_u_.

Finally, we note that the folded/unfolded equilibrium that defines ΔG_u_ has a different relationship to function in globular proteins than in intrinsically disordered (ID) proteins: Globular proteins *must* be folded to function. In contrast, some ID proteins may never adopt a regular structure; “function” is a property of the ensemble of unfolded conformations rather than of folded conformations. In other ID proteins, ligand binding and protein folding are tightly coupled (125), making it extremely difficult to experimentally dissect the two parameters. For example, in the ID transactivation domain of CIITA, substitutions at two rheostat positions show a correlation between transcriptional activation and intrinsic structural propensities, consistent with substitution effects on coupled folding and binding (35).

### “Stability rheostat positions” modulate ΔG_u_

**Figure 8:**
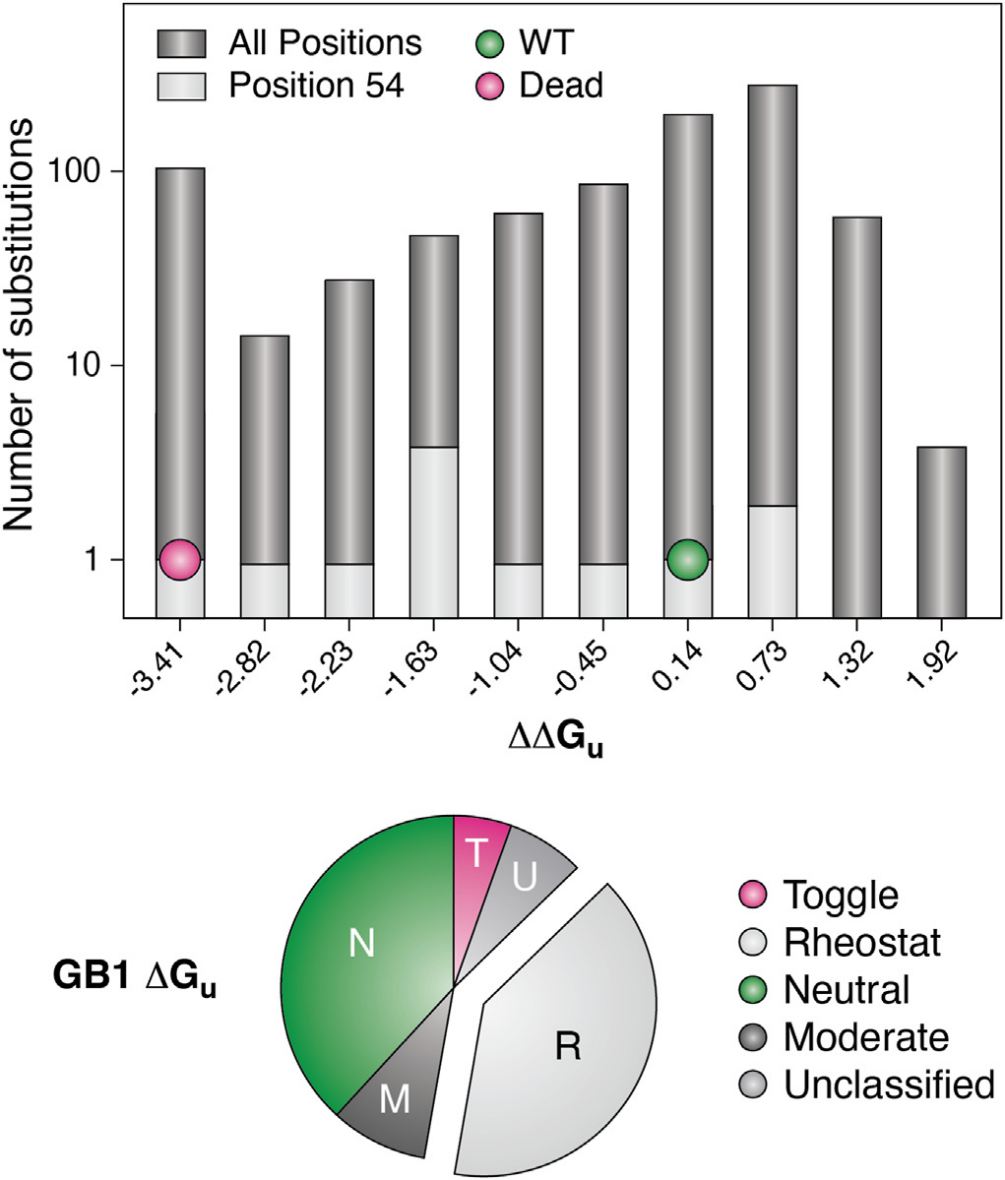
**SSM in GB1 leads to a wide range of stabilities and reveals numerous rheostat positions.***Top*, data from (115) were used to create histograms of (*dark gray*) stability outcomes for all 933 GB1 variants (including both quantitatively measured and “dead” variants, which assigned a ΔΔG_u_ of −4) and (*light gray*) values for 18 amino acid substitutions at position 54. The bins corresponding to WT (*green*; 0.14) and dead (*magenta*; −3.41) are indicated with *circles*. *Bottom*, RheoScale analyses (17) were carried out using histograms for each set of variants at all GB1 positions; calculations of rheostat and toggle scores used ten bins; calculations for neutral scores used five bins to encompass error in the wild-type measurements (as described in (31)). In addition to rheostat (R), toggle (T) and neutral (N) substitution outcomes, a few positions show “moderate” effects (M) on stability; that is, most substitutions showed significant differences from wild-type but did not meet the threshold of sampling at least half of the stability range. Substitution outcomes for a few positions could not be classified (U).

Analogous to functional rheostat positions, “stability rheostat positions” were hypothesized in 2016 (53). At such positions, effects on stability would vary in an amino acid-dependent manner, and values of ΔG_u_ would span a wide range. Subsequently, Matthews *et al.* carried out deep mutational scanning studies on a region in a TIM barrel known to alter stability in three indole-3-glycerol phosphate synthase isozymes (42). Several of these positions had rheostatic outcomes (17), although effects on stability and function were likely to be intertwined in this assay. More convincingly, Mayo *et al.* combined SSM with a high-throughput, quantitative approach for measuring ΔG_u_ in the B1 domain of Streptococcal Protein G (GB1; (115)). Forty percent of GB1’s positions had rheostatic substitution outcomes on stability (Fig. 8); substitutions at the strongest rheostat position sampled nearly the full range of observed stabilities.

Mayo *et al.* also noted that, except for proline and glycine substitutions, some positions tolerated a wide range of side chains with little effect on ΔG_u_, whereas other positions did not. Thus they concluded that a position’s location on the structure was more important to governing stability than the chemistry of the amino acid substitution (115). Likewise, positions associated with stability changes in the TIM barrels study (42) also showed a structural pattern, with the loops enriched for rheostat positions and one side of the beta-barrel enriched for toggle positions (17). These location-based findings reinforce the value of assessing each *position’s* structural (or functional) role instead of considering separate roles for each of the 20 possible acid side chains with which it can be substituted.

Going forward, it will be interesting to determine whether the presence of stability rheostat positions and the distance correlation observed in GB1 generalize to other proteins. Another consideration is that, with 56 amino acids, GB1 is fairly small, and single substitutions alter a large percent of its bonds (which additively contribute to its stability; described in (126, 127, 128, 129)). As such, it could be easier for different substitutions at a single position to widely sample a range of stabilities, allowing stability rheostat positions to exist. In contrast, substitutions in larger proteins—which alter a smaller percent of the bonds that contribute to stability—might be limited to small effects on protein stability, precluding the presence of stability rheostat positions. To tune the stability of larger proteins over a large range, simultaneous substitutions at multiple positions might be required. Nonetheless, even oligomeric proteins may be susceptible: a 2024 study found that substitutions at a nonconserved, interfacial position in a trimeric chloramphenicol acetyltransferase (291 aa monomer) had rheostatic effects on the midpoint for thermal denaturation (T_m_), with substitutions sampling a range of nearly 30 °C (130).

Finally, future studies of globular proteins should consider whether stability and functional rheostat positions comprise separate sets of positions or are inter-twined. For example, it is not yet known whether (i) the GB1 stability rheostat positions play any role in GB1 function or (ii) the functional rheostat positions identified in various model proteins (Table 1) also alter ΔG_u_. Interestingly, for enzymes, there is a widespread expectation of a “stability/activity tradeoff” (*e.g.*, (131, 132, 133, 134)). However, Whitehead *et al.* conducted SSM for two enzymes and compared each variant’s effect on (i) its cellular function, as a proxy for protein function and (ii) the amount of folded protein recognizable by antibody in a yeast display assay, as a proxy for protein stability. These studies found a range of “stability/activity” relationships (21). As such, the function/stability relationship does not appear generalizable within or among proteins, and we do not expect this to be the case for rheostat positions.

Thus, predictions about substitution outcomes will need to account for both changes in function and stability. An early prototype of such a combined algorithm using a simple biochemical equation is described in (120) and a recent advancement using machine learning to analyze high-throughput functional data, patterns of evolutionary change, and predicted stability changes is described in (135). Ideally, in the future, parallel stability and functional experiments will be generated for large numbers of model proteins to further generalize such models.

## Conclusion

### Tuneability—a new conceptual framework for understanding the protein structure-function relationship?

**Figure 9:**
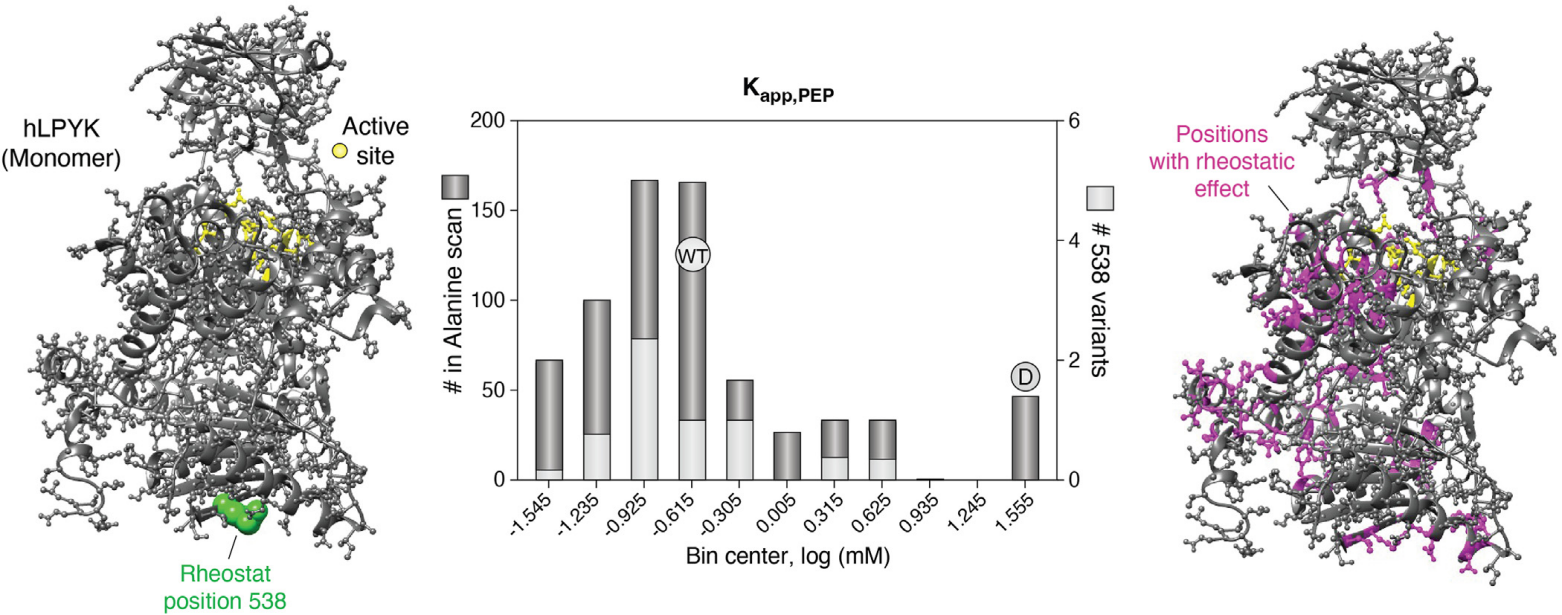
**Functional tunability.** The structure on the *left* shows one monomer of tetrameric hLPYK. *Gray side chains* are used to show the locations of 427 positions that were substituted with alanine (59); *green spacefilling* is used to show the location of rheostat position 538, which was substituted with 13 additional amino acids (14 total variants; (33)). The enzyme active site is shown in *yellow*; many of these positions were also substituted with alanine. The center plot depicts a histogram of the apparent binding affinities for substrate, K_app-PEP_, (which is mediated by the active site) measured for each of these two sets of variants. Note that 14 substitutions at rheostat position 538 (*light gray bars*) sampled nearly the exact same range of change as the whole-protein alanine scan (*dark gray bars*). The structure on the *right* shows the >80 positions (*magenta*) that are predicted to have rheostatic effects on K_app-PEP_ (44); other side chains are colored the same as on the *left*. This illustration was created in UCSF Chimera (137) from the hLPYK PDB 4IMA (68).

In hindsight, perhaps the existence of functional rheostat positions should have been anticipated from earlier studies. Their effects are almost certainly manifest in various “scanning” data sets, which have revealed that some protein functions are highly tunable by single amino acid substitutions. For example, when 427 of 543 positions were substituted in an alanine scan of hLPYK, values of K_app-PEP_ fully sampled the >200-fold, observed range (Fig. 9; (59)). Rheostat positions are but a special case of this substitution behavior—the same range of functional outcomes is available *via* substitutions at just a single position. For example, in hLPYK, the same 200-fold range of functional range was well-sampled by just 14 substitutions at a single rheostat position (33), and >70 hLPYK positions appear to be rheostat positions for K_app-PEP_ (Fig. 9; (44)).

Thus, we conclude here by considering how the existence of rheostat positions might give us new insights into the general relationship between protein sequence and function: Rather than attempting to formulate separate structural mechanisms that explain how each substitution at each rheostat position modulates function, it is intriguing to consider whether a single mechanistic framework can provide an explanation for many of the changes. For example, the substitutional-modulation of an anisotropic dynamic network may provide one global perspective for describing altered functional outcomes (*e.g.*, (37, 136)).

Understanding the molecular mechanisms by which substitutions at rheostat positions give rise to a wide range of responses will be critical for advancing personalized medicine and protein engineering. Future studies to identify the locations of rheostat positions will enable experimental studies that focus on these interesting and widespread positions. Given the wide range of complex functional outcomes that arise from substitutions at rheostat positions—including their potential for enhancing function, simultaneous effects on multiple biochemical parameters, effects on ligand specificity, their independence of side chain chemical “similarities”, and their long-range through-protein effects—it is imperative to understand the biophysical roots of these intriguing protein positions.

## Conflict of interest

The authors declare that they have no conflicts of interest with the contents of this article.
